# CircRNA Identification and CircRNA–miRNA–mRNA Network in *Cynoglossus semilaevis* Sexual Size Dimorphism

**DOI:** 10.3390/biology11101451

**Published:** 2022-10-02

**Authors:** Zhihong Gong, Rui Shi, Songlin Chen, Na Wang

**Affiliations:** 1 Laboratory for Marine Fisheries Science and Food Production Processes, Qingdao National Laboratory for Marine Science and Technology, Yellow Sea Fisheries Research Institute, Chinese Academy of Fishery Sciences, Qingdao 266071, China; 2 College of Marine Life Sciences, Ocean University of China, Qingdao 266100, China; 3 College of Fisheries and Life Science, Shanghai Ocean University, Shanghai 201306, China; 4 Key Laboratory for Sustainable Development of Marine Fisheries, Ministry of Agriculture, Qingdao 266071, China

**Keywords:** Chinese tongue sole, sexual size dimorphism, circular RNA, ceRNA

## Abstract

**Simple Summary:**

Chinese tongue sole (*Cynoglossus semilaevis*) typically displays female-biased sexual size dimorphism (SSD), but its epigenetic regulatory mechanisms are poorly understood, especially the role of circRNAs. To explore the function of circRNAs in Chinese tongue sole SSD, we firstly identified differentially expressed circular RNAs (DE circRNAs) in female, male, and pseudo-male *C. semilaevis*. Secondly, the ceRNA network containing DE circRNAs, miRNAs, and mRNAs in the three genders was constructed. Among the ceRNA network, several circRNAs such as novel_circ_004374 and novel_circ_014597 may regulate *hipk2* expression by sponging miR-130-x. It is also worth exploring whether or how novel_circ_008696 regulates *setd2* by binding to novel-m0387-3p. The present study provided the cirRNA and its ceRNA network that potentially regulate *C. semileavis* female-biased SSD for the first time.

**Abstract:**

Sexual size dimorphism (SSD), which is the sexual differences in body size, has been widely reported in various species including fishes. For Chinese tongue sole (*Cynoglossus semilaevis*), a flatfish exhibiting typically female-biased SSD, little is known for its epigenetic regulation mechanism, especially the role of circRNAs. Here, we identified the differently expressed abundances of circRNAs in females, males, and pseudo-males to explore the potential functions of circRNAs in Chinese tongue sole SSD. In total, 14,745 novel circRNAs were screened, among which 1461 DE circRNAs were identified from the brain, gonad, liver, and muscle in female, male, and pseudo-male individuals. The ceRNA network was subsequently constructed, including 10 circRNAs, 26 mRNAs, and 11 miRNAs. These DE mRNAs were mainly related to the mRNA surveillance pathway, metabolic pathways, and cellular senescence. Importantly, the ceRNA network has revealed that several circRNAs such as novel_circ_004374 and novel_circ_014597 may regulate *homeodomain interacting protein kinase 2* (*hipk2*) expression by sponging miR-130-x. It is also worth exploring whether or how novel_circ_008696 regulates *SET Domain Containing 2, histone lysine methyltransferase* (*setd2*), which in turn affects the epigenetic patterns of different sexual individuals. The present study not only enriches the knowledge on the potential roles of circRNA in the physiological process, but also provides new clues for the explanation of fish SSD. In future studies, the precise function and involvement of circRNAs in female-biased SSD will require more efforts.

## 1. Introduction

Many species have evolved to exhibit female and male body size differences, such as frogs, lizards, birds, and bats, and this sexual size dimorphism (SSD) may increase their survival advantage in the wild [1,2,3,4]. It has been reported that more than 600 kinds of fish species exhibit female or male-biased SSD [5], which would cause growth disadvantages for male or female individuals, restricting in this way the sustainable development of fish aquaculture [6]. For Chinese tongue sole (*Cynoglossus semilaevis*), a typically female-biased SSD flatfish in aquaculture [7], its females grow 2–4 times faster than the males [8,9]. Moreover, the environment factors including high temperatures often cause female-to-male sex reversal, and the pseudo-males exhibit physiological characteristics and growth performances similar to the males [10]. Thus, a low proportion of females has affected the work of *C. semilaevis* farmers and increased the cost of aquaculture [11]. 

Previous studies have focused on the sexually dimorphic expression in *C. semialevis* female and male individuals at the gene, pathway, and transcriptomic levels [8,12,13,14]. The classical growth axis genes exhibit significantly differentially expressed patterns in different sexes [8,13,14], as well as multiple pathways containing steroid biosynthesis, MAPK signaling pathway, and glycolysis. Recently, whole transcriptome and DNA methylation analyses not only reveal that cell growth and death-related pathways are implicated in *C. semilaevis* female-biased SSD, but also identify lncRNA–miRNA–mRNA networks that participate in SSD by potentially regulating growth-related pathways [15,16]. The circular RNAs (circRNAs), firstly discovered as non-coding RNAs with covalently closed-loop structures [17,18], have been identified in many species including mammals, nematodes, plants, and archaea [19,20,21,22,23]. CircRNA plays important roles in many biological and pathophysiological processes including immune, metabolism, skeletal muscle and fat tissue growth, follicle development, nerve system development, cell proliferation, and diseases [24,25], by decoying miRNAs, binding to protein, and translating into peptides [26,27,28,29,30,31,32,33,34,35]. In teleosts such as the large yellow croaker (*Larimichthys crocea*), the grass carp (*Ctenopharyngodon idellus*), and the Nile tilapia (*Oreochromis niloticus*), circRNAs have been identified to be involved in growth, immunity, and heat stress [36,37,38,39,40,41]. In *C. semilaevis*, circRNA has been reported to regulate sex determination and differentiation [42,43,44]. However, there is no research on the roles of circRNA on Chinese tongue sole SSD.

Therefore, the present study aimed to illustrate the circRNAs that might regulate *C. semilaevis* SSD by whole transcriptomic analysis of four important tissues including brain, liver, gonad, and muscle from female, male, and pseudo-male individuals. The competing endogenous RNA (ceRNA) network of DE circRNAs was constructed. Moreover, the enriched GO terms and pathways were analyzed to reveal the potential effect of circRNA on *C. semilaevis* SSD.

## 2. Methods

### 2.1. Fish Treatment, Samples Collection, and Ethics Statement

The 1.5-year-old Chinese tongue soles used in the experiment were raised in Haiyang Yellow Sea Fisheries Limited Company (Yantai, China). The fish were anesthetized with MS-222 (Sinopharm, Shanghai, China) before dissection to reduce pain. The experiment was approved by the Animal Care and Use Committee at the Chinese Academy of Fishery Sciences (Approval number: YSFRI-2022024). The fish-rearing conditions were as follows: 22–26 °C, 30‰ salinity, 8 h of light and 16 h of darkness. Following the PCR protocol in a previous study [45], genetic sex of males and pseudo males were identified by primers sex-F and sex-R, which amplify 169 and 134 bp fragments from pseudo males, and only 169 bp fragment from males (Figure S1). Four tissues including the brain, liver, gonad, and muscle, were sampled from females, males, and pseudo males (for each gender, n = 9). Three replicate samples were combined into one sample, resulting in 36 samples named FB (female brain) (1–3), FG (female gonad) (1–3), FL (female liver) (1–3), FM (female muscle) (1–3), MB (male brain) (1–3), MG (male gonad) (1–3), ML (male liver) (1–3), MM (male muscle) (1–3), PMB (pseudo male brain) (1–3), PMG (pseudo male gonad) (1–3), PML (pseudo male liver) (1–3), and PMM (pseudo male muscle) (1–3). The collected tissues were immediately stored in liquid nitrogen. 

### 2.2. RNA Extraction and High-Throughput Sequencing

Total RNA was extracted with TRIzol reagent (Invitrogen, Carlsbad, CA, USA) following the manufacturer’s protocol and RNA quality was evaluated by Agilent 2100 Bioanalyzer (Agilent Technologies, Palo Alto, CA, USA) and agarose gel electrophoresis. RNAs with RIN > 7.0 were submitted for library construction. Ribosomal RNAs (RNAs) were removed to enrich mRNAs and ncRNAs by Ribo-ZeroTM Magnetic Kit (Epicentre, Madison, WI, USA). The enriched ncRNA was fragmented into short fragments using fragmentation buffer and reverse transcribed into cDNA by using VAHTS Total RNA-seq (H/M/R) Library Prep Kit for Illumina (Vazyme, Nanjing, China). The segments’ purification was conducted by QIAQuick PCR Extraction Kit (QIAGEN, Venlo, the Netherlands) and the second strand degradation was performed by Uracil-N-Glycosylase. The fragment was subsequently amplified with 15 cycles, and the cDNA quality and quantity were assessed on Agilent 2100 Bioanalyzer (Agilent Technologies, Palo Alto, CA, USA) and Qubit 4.0 fluorometer (Thermo Fisher Scientific, Waltham, MA, USA). The cDNA library was sequenced by Illumina HiSeqTM 4000 in Gene Denovo Biotechnology Co. (Guangzhou, China). Raw reads were analyzed with the fastp software (version 0.20.1) [46] to filter low-quality data and obtain clean reads. The alignment tool bowtie2 (version 2.3.2) [47] was used to align the clean reads to remove the aligned ribosome reads without allowing mismatches, and the retained unmapped reads were used for subsequent analysis. HISAT2 (version 2.1.1) software [48] was used to align the reference *C. semilaevis* genome (NCBI GCA_000523025.1, Cse_v1.0) to construct a reference set of splice sites, and find_circ [20] was employed to identify circRNAs. DE circRNAs were assigned as |log2fold change| ≥ 1 and *p* value < 0.05 by edgeR (version 3.12.1) [49] package.

### 2.3. circRNA–miRNA–mRNA Association Analysis

A circRNA–miRNA–mRNA regulatory network was built with DE circRNAs, DE miRNAs, and DE mRNAs. The ceRNA network between DE lncRNAs, DE miRNAs, and DE mRNAs data was analyzed in our previous study [16]. The miRNA targets were predicted using TargetScan software (version 8.0). The correlation of DE mRNA–DE miRNA or DE circRNA–DE miRNA expression was evaluated by Spearman Rank correlation coefficient (SCC) < −0.7. Pearson correlation coefficient (PCC) was used to evaluate the correlation of DE circRNA–DE mRNA expression [50]. The circRNA–mRNA pairs with PCC > 0.9 were selected as co-expressed negatively miRNA pairs. The common miRNA sponges with *p* values < 0.05 were set as significant. The Sankey diagram and circRNA expression heatmap were made with OmicShare tool, a free online platform for data analysis (www.omicshare.com/tools, accessed on 6 March 2022). The Venn diagrams were created using the VennDiagram (version 1.7.3) package.

### 2.4. Function Enrichment Analysis 

To predict the biological functions DE circRNAs, Gene Ontology (GO) (http://geneontology.org/, accessed on 6 March 2022)and Kyoto Encyclopedia of Genes and Genomes (KEGG) (https://www.kegg.jp/, accessed on 6 March 2022) pathway analyses were conducted for the ceRNAs-relevant mRNAs. 

### 2.5. RT-PCR and qRT-PCR 

Total RNA was extracted from the tissues by TRIzol reagent (Invitrogen, Carlsbad, CA, USA) following the manufacturer’s protocol. Reverse transcription was performed to synthesize cDNA with random primers using PrimeScript^TM^ RT Reagent Kit (Takara, Shiga, Japan). Real-time reverse transcription PCR (qRT-PCR) was performed using SYBR GREEN qPCR Super Mix (Invitrogen, Carlsbad, CA, USA) on 7500Fast Real-Time PCR System (Applied Biosystems, Foster City, CA, USA). A 20 μL qRT-PCR reaction was conducted at 95 °C for 30 s, 40 cycles at 95 °C for 5 s, 60 °C for 33 s, with the next stage at 95 °C for 15 s, 60 °C for 60 s. According to a previous method [51], primers were designed to span the cleavage site to discriminate between linear and circular RNA transcripts. Sequences of all primers used in qRT-PCR are presented in Table 1. Detection of the length of PCR products by agarose gel electrophoresis and Sanger DNA sequencing of reverse transcription PCR (RT-PCR) products was used to demonstrate the presence of circRNAs. The qRT-PCR data were analyzed by the 2^−ΔΔ*C*T^ method [52] and the data obtained from qPCR were performed by two-way ANOVA test using GraphPad Prism 9 (version 9.3.0).

## 3. Result

### 3.1. Overview of circRNA Identification in Chinese Tongue Sole 

**Figure 1:**
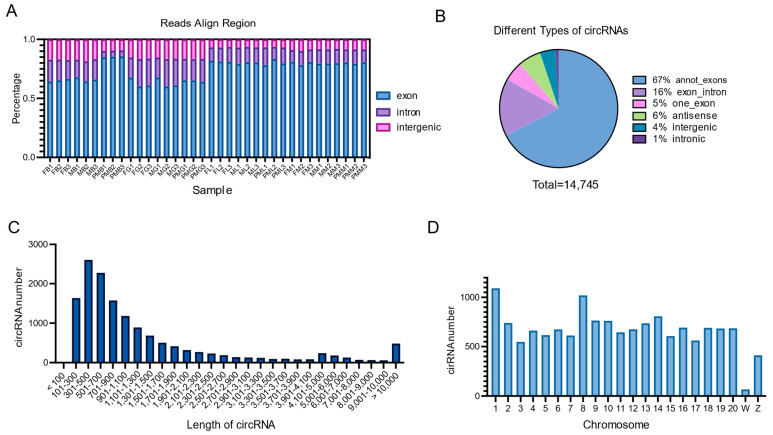
Identification and analysis of circRNAs. (**A**) CircRNA reads align regions in different samples. (**B**) Different types of circRNAs. (**C**) Length of circRNAs. (**D**) CircRNAs numbers of different Chromosomes.

In total, 14,745 novel circRNAs were identified from the brain, liver, muscle, and gonad tissues of male, female, and pseudo-male Chinese tongue soles. Over 70% of the reads were obtained by sequencing in the exon region (Figure 1A), and the majority of cirRNA types were annot_exons (67%) and exon_intron (16%) (Figure 1B). The length of most circRNAs (85%) ranged from 100 nt to 2500 nt and at 11% from 2501 nt to 10,000 nt (Figure 1C). Most of the chromosomes, except for the W chromosome, produce more than 400 kinds of circRNAs (Figure 1D). 

### 3.2. Differentially Expressed circRNAs 

**Figure 2:**
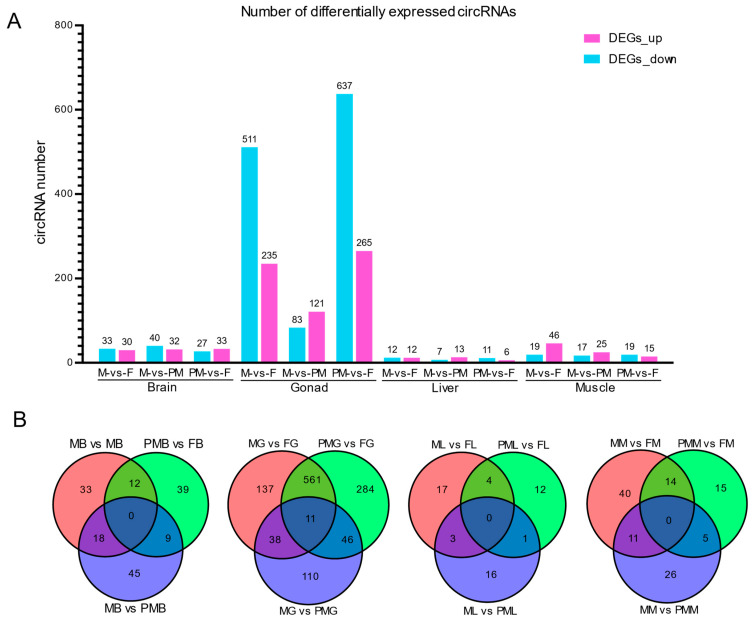
Identification and characterization for DE circRNAs in different sexual *C. semilaevis*. (**A**) Basic difference analysis histogram of DE circRNAs from the brain, gonad, liver, and muscle in female, male, and pseudo-male individuals. (**B**) Venn diagrams of DE circRNAs.

Among all screened circRNAs, we identified 1461 differentially expressed circRNAs (DE circRNAs) through pairwise comparisons of four different tissues in female, male, and pseudo-male fish (Figure 2). DE circRNAs were mainly identified in gonads, with 1187 differentially expressed circRNAs (Figure 2A). In the Venn diagram of the gonad DE circRNAs, there are 561 genes in the largest intersection (Figure 2B), which are the differential genes shared by males and pseudo males among females, indicating that males and pseudo males have similar circRNA expression patterns.

### 3.3. Verification of Differentially Expressed circRNAs 

**Figure 3:**
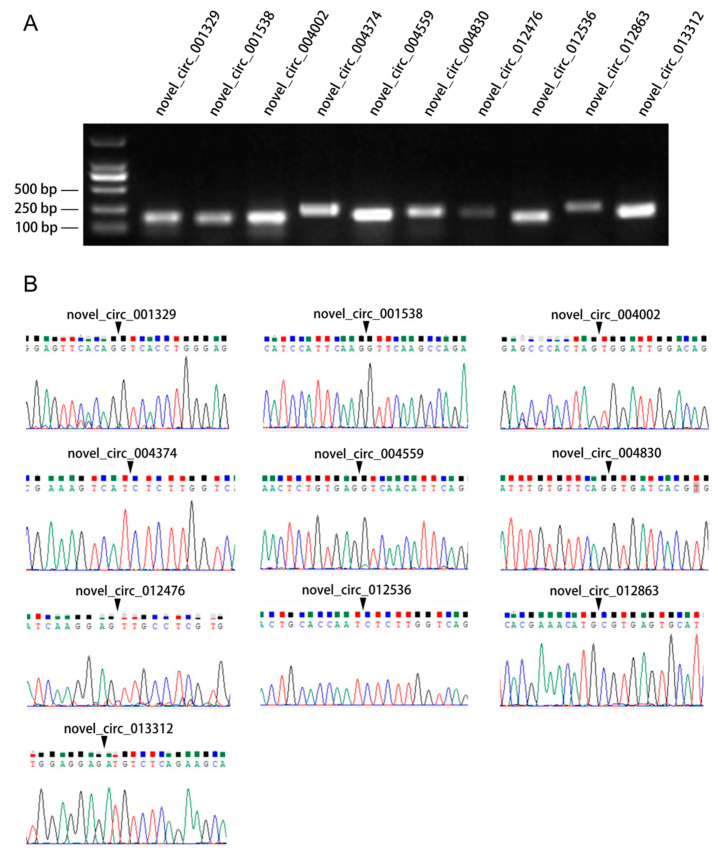
The agarose gel electrophoresis analysis and sequencing for novel circRNAs. (**A**) Agarose gel electrophoresis analysis demonstrating the length of circRNAs. (**B**) Sequence verification for circRNAs. Arrows show the splice junction of circRNAs.

To verify the reliability of circRNAs derived from RNA-seq, we selected 10 differentially expressed circRNAs (novel_circ_001329, novel_circ_001538, novel_circ_004002, novel_circ_004374, novel_circ_004559, novel_circ_004830, novel_circ_012476, novel_circ_012536, novel_circ_012863, novel_circ_013312), and amplified by RT-PCR primers (Table 1). The results of agarose gel electrophoresis showed the length of the PCR products (Figure 3A), as expected from transcriptome sequencing. The PCR products were sequenced by Sanger sequencing to confirm the junction sites, as well as upstream and downstream sequences around the splice junctions, shown in Figure 3B. These all support the presence of circRNAs.

**Figure 4:**
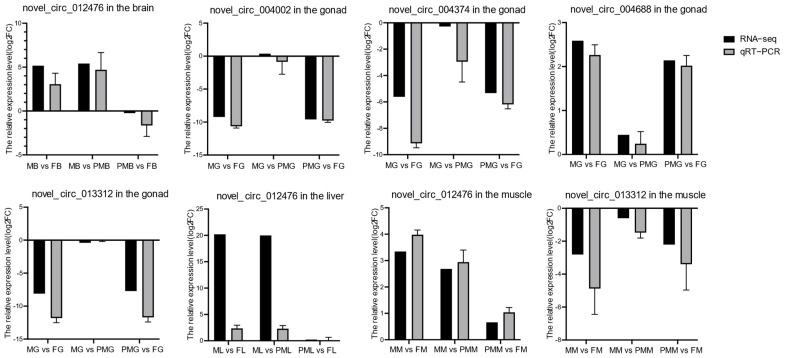
The quantitative PCR (qPCR) verification for the expression patterns of circRNAs. Gray and black legends represent qPCR and RNA-seq results, respectively.

Eight circRNAs including novel_circ_012476, novel_circ_004002, novel_circ_004374, novel_circ_013312, novel_circ_004688, novel_circ_012476, and novel_circ_013312 were selected for qRT-PCR validation. As a result, the expression trends of these cirRNAs by qRT-PCR corresponded well with the patterns of high-throughput sequencing (Figure 4).

### 3.4. Differentially Expressed circRNAs ceRNA Network

**Figure 5:**
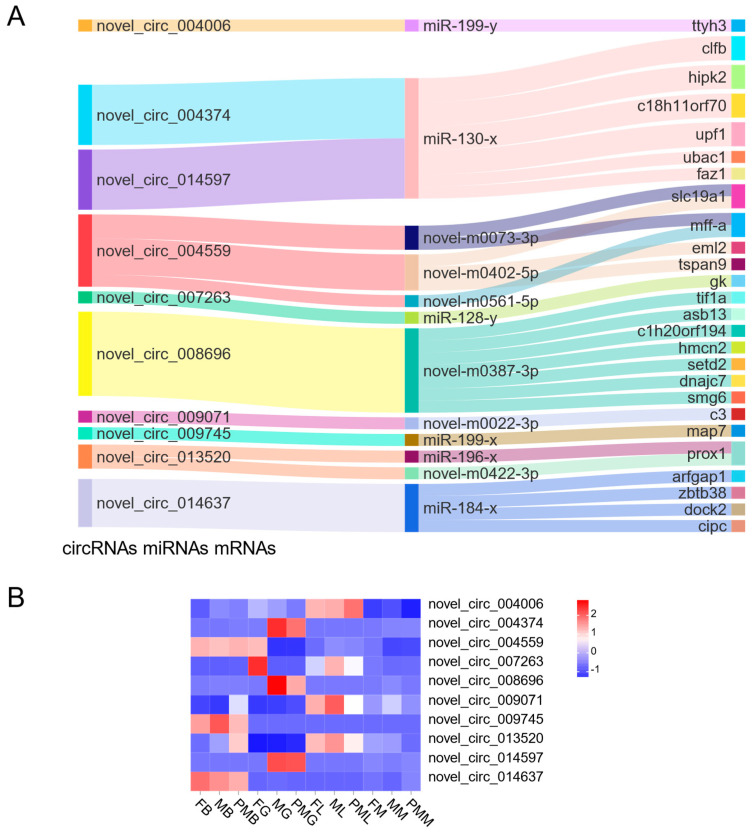
Functional circRNA–miRNA–mRNA regulatory module and the different expression of circRNAs in males, females, and pseudo males. (**A**) The circRNAs, miRNAs, and mRNAs are on the left, middle, and right, respectively. (**B**) Heatmap of the different expression of circRNAs in gonads, brains, muscles, and livers of males, females, and pseudo males.

CircRNAs may bind to sequence-complementary miRNAs and affect the post-transcriptional regulation of miRNAs on their target genes. To assess the underlying functions of DE circRNAs as ceRNAs, we constructed the ceRNA network for the differentially expressed circRNAs (Figure 5A). A total of 10 circular RNAs competed with 26 mRNAs for binding to 11 miRNAs, of which 6 circular RNAs (novel_circ_004374, novel_circ_004559, novel_circ_007263, novel_circ_008696, novel_circ_009071, and novel_circ_014597) were DE circular RNAs in the gonad (Figure 5). Novel_circ_009071 and novel_circ_013520 were highly expressed in pseudo-male brains (Figure 5B). Novel_circ_004559 was highly expressed in female muscle. Novel_circ_007263 were was expressed in male liver.

### 3.5. Biological Functions of ceRNAs-Relevant mRNAs

In order to predict the potential functions of DE circRNAs, GO and KEGG pathway analysis for the ceRNAs-relevant mRNAs were performed. GO analysis of ceRNAs-associated mRNAs revealed that they were represented in metabolic, development, and others biological processes (Figure S2A). KEGG pathway enrichment showed that the ceRNAs-associated mRNAs were involved in cellular senescence, mRNA surveillance pathway, metabolic pathways, and other pathways (Figure S2B).

## 4. Discussion

Chinese tongue soles have a female heterogametic (WZ female, ZZ male) sex chromosome system and show obviously female-biased SSD [15]. As an important regulator for gene expression [53], circRNAs have been identified to be implicated in myogenesis, skeletal muscle and fat tissue growth, follicle development, and sperm motility [54,55,56,57]. Herein, we identified the different expression abundances of circRNAs in males, females, and pseudo males to examine the potential roles of circRNAs in *C. semilaevis* SSD.

In our study, a total of 14,745 novel circRNAs were identified, with most circRNAs derived from exon or exon_intron, with the exception of only 4% intergenic circRNAs (Figure 1B). From the perspective of chromosome distribution, the number of circRNAs in the W chromosome is fewer than in the Z and autosomal chromosomes (Figure 1D). Given that most circRNAs are generally produced from pre-mRNA back-splicing [24], the reason for there being few circRNAs in the W chromosome may be attributed to the fact that the W chromosome of *C. semilaevis* has the least number of functional genes (317), accounting for 17–34% of other chromosomes [9]. As in previous studies [43,57,58,59], the *C. semilaevis* circRNAs exhibited obvious tissue and sex-specific expression patterns, with gonads showing the largest number of differentially expressed circRNAs in different genders. 

The lengths of most circRNAs (85%) ranged from 100 nt to 2500 nt in the present study, which is similar to grass carp circRNAs [60], while 78% of the circRNAs identified in the Japanese flounder were longer than 5000 nt [37]. In mammals, the length of the majority of circRNAs is less than 3500 nt in rats [58] and longer than 3000 nt in pigs [55]. These data suggest that the lengths of circRNAs might be affected by factors such as species, tissues, and the length of the gene itself.

CircRNAs have been involved in cellular and physiological regulation by decoying miRNAs [27,28,29,61]. The ceRNA network showed that 10 circRNAs competed with 26 mRNAs for binding to 11 miRNAs. The enrichment of ceRNA-relevant mRNAs showed that cellular senescence, mRNA surveillance pathway, and metabolic pathways were involved. Noticeably, cellular senescence was also identified from the brown module, the negative SSD-related module in previous analyses based on the same branch of samples [15]. 

*Homeodomain interacting protein kinase 2* (*hipk2*), a gene derived from a cellular senescence pathway, was highly expressed in male and pseudo-male gonads. *Hipk2* is involved in transcription control, cellular apoptosis, and cell cycle regulation [62,63]. Importantly, studies have reported that *hipk2* may inhibit cell growth [64]. In the present study, novel_circ_004374 and novel_circ_014597 may modulate *hipk2* by sponging to the miR-130. Interestingly, more than 30 lncRNAs were also predicted to be involved in *hipk2* regulation by sponging miR-130 [16]. The detailed molecular mechanism needs further investigation.

Novel_circ_008696 was found to compete with *SET Domain Containing 2, histone lysine methyltransferase* (*setd2*), for binding to novel-m0387-3p. Setd2 is the main enzyme generating H3K36me3 (Histone 3 lysine 36 trimethylation), which provides docking sites for many chromatin regulators including DNA (cytosine-5)-methyltransferase 3A/B (dnmt3a/b) [65,66]. Interestingly, the present study and our previous transcriptome data have revealed that *setd2* and *dnmt3a/b* exhibited male and pseudo-male biased expression levels in the gonad [15]. In the present study, novel_circ_008696, a competitor for *setd2*, was also expressed in higher levels in male and pseudo-male gonads. The overexpression of *setd2* inhibited osteosarcoma cell growth by suppressing Wnt/β-catenin signaling [67]. Its role in growth regulation remains unknown. Thus, it is interesting to study whether or how novel_circ_008696 regulates *setd2*, which in turn affects the epigenetic levels in different sexual individuals. This information would be valuable for understanding the roles of non-coding RNAs in female-biased SSD. 

In mammals, birds, and teleosts, it was reported that circRNA plays important roles in muscle growth regulation [41,68,69,70]. Moreover, there are also studies addressing the involvement of circRNAs in the ovary development and sex determination for birds and teleosts [43,44,71]. Therefore, the present study not only enriches the potential roles of circRNA in physiological processes, but it also provides new clues for the explanation of fish SSD. Nevertheless, the circRNA–miRNA–mRNA regulatory networks require more research to elucidate the ceRNA mechanisms in Chinese tongue sole SSD. 

## 5. Conclusions

The present study proved that cirRNA and its ceRNA network potentially regulate *C. semileavis* female-biased SSD for the first time. A total of 1461 DE circRNAs have been identified from the brain, gonad, liver, and muscle in female, male, and pseudo-male individuals. Importantly, the ceRNA network has revealed that several circRNAs such as novel_circ_004374 and novel_circ_014597 may regulate *hipk2* expression by sponging miR-130-x. In addition, whether or how novel_circ_008696 regulates *setd2* by binding to novel-m0387-3p was addressed. In future studies, the examination of the precise function and involvement of circRNAs in female-biased SSD requires more efforts. Only then will we be able to utilize a circRNA- or miRNA- based genome editing tool for sustainable aquaculture production.

## Figures and Tables

**Table 1:** Primer sequences in this study.

Primer	Primer Sequence (5′-3′)
sex-F	CCTAAATGATGGATGTAGATTCTGTC
sex-R	GATCCAGAGAAAATAAACCCAGG
β-actin-F	GCTGTGCTGTCCCTGTA
β-actin-R	GAGTAGCCACGCTCTGTC
nc1329-F	GGACATCGTTTCCTCCATC
nc1329-R	TGCTTCCTCTTCTGTAGCCC
nc1538-F	TGTGTCTTGGAGAAGGACTG
nc1538-R	TTTCAGCGAACTCTGGCT
nc4002-F	CTTGTGGATGAGATTCTGACTG
nc4002-R	ACTTGGAAACAGGCAGCAC
nc4374-F	GCTATTGATGACCCAGTGAA
nc4374-R	CACCACCAAACGGAACTT
nc4559-F	GCAGGCATTTGTGATGTCA
nc4559-R	GGCTTATTGGTCAGATTGTGTC
nc4830-F	GCCGCTGTTCGGATTATT
nc4830-R	ACAACGACAGGACAATGGG
nc12476-F	GCGTCTTTGACCACCGTAT
nc12476-R	TGTTGACCATTCGGAGGA
nc12863-F	CACATACTTCTTACGCACGAC
nc12863-R	GAATCATCTCCTCCTCCAAG
nc13312-F	CATCCAGAAGTAATGACCATCC
nc13312-R	AGAAGTCCTCCTATGACCTGC

## Data Availability

The datasets presented in this study can be found in the NCBI SRA repository with accession number PRJNA743138 (https://www.ncbi.nlm.nih.gov/bioproject/PRJNA743138/) (Accessed on 10 September 2021).

## Supplementary Materials

The following supporting information can be downloaded at: https://www.mdpi.com/article/10.3390/biology11101451/s1, Figure S1. The sex identification for male and pseudo male individuals; Figure S2. The GO (A) and KEGG (B) analysis of the DE mRNAs in the circRNA-miRNA-mRNA network.
