# Supplementary material for: CircRNA Identification and CircRNA–miRNA–mRNA Network in Cynoglossus semilaevis Sexual Size Dimorphism
Source: Biology (Basel). 2022 Oct 2;11(10):1451. doi: 10.3390/biology11101451 (PMC9598273; doi:10.3390/biology11101451)

The supplementary file for

CircRNA identification and circRNA-miRNA-mRNA network in *Cynoglossus semilaevis* sexual size dimorphism

Zhihong Gong[a, b], Rui Shi[a, c], Songlin Chen[a, d], Na Wang[a, d*]

[a] Laboratory for Marine Fisheries Science and Food Production Processes, Qingdao National Laboratory for Marine Science and Technology, Yellow Sea Fisheries Research Institute, Chinese Academy of Fishery Sciences, Qingdao 266071, China
[b] Ocean University of China, Qingdao 266100, China
[c] College of Fisheries and Life Science, Shanghai Ocean University, Shanghai 201306, China
[d] Key Laboratory for Sustainable Development of Marine Fisheries, Ministry of Agriculture, Qingdao 266071, China
*Correspondence: Prof. Na Wang
Yellow Sea Fisheries Research Institute
Chinese Academy of Fishery Sciences
106 Nanjing Road
Qingdao 266071, China
Tel: (+) 86-532-85831605;
Fax: (+) 86-532-85811514;
Email: wangna@ysfri.ac.cn

Figure S1. The sex identification for male and pseudo male individuals.
Figure S2. The GO (A) and KEGG (B) analysis of the DE mRNAs in the circRNA-miRNA-mRNA network.

Figure S1. The sex identification for male and pseudomale individuals.

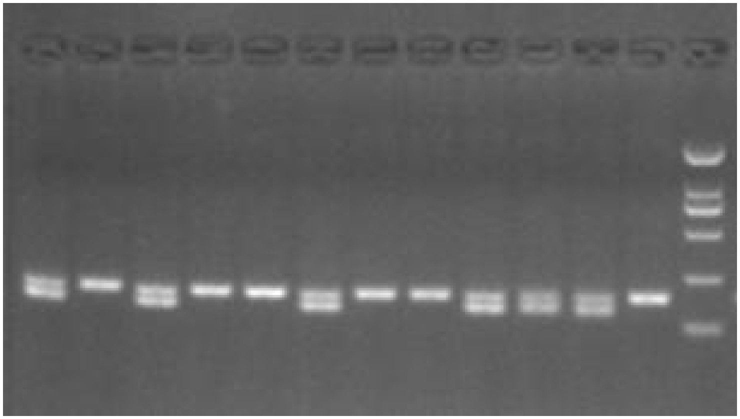

Figure S2. The GO (A) and KEGG (B) analysis of the DE mRNAs in the circRNA-miRNA-mRNA network.

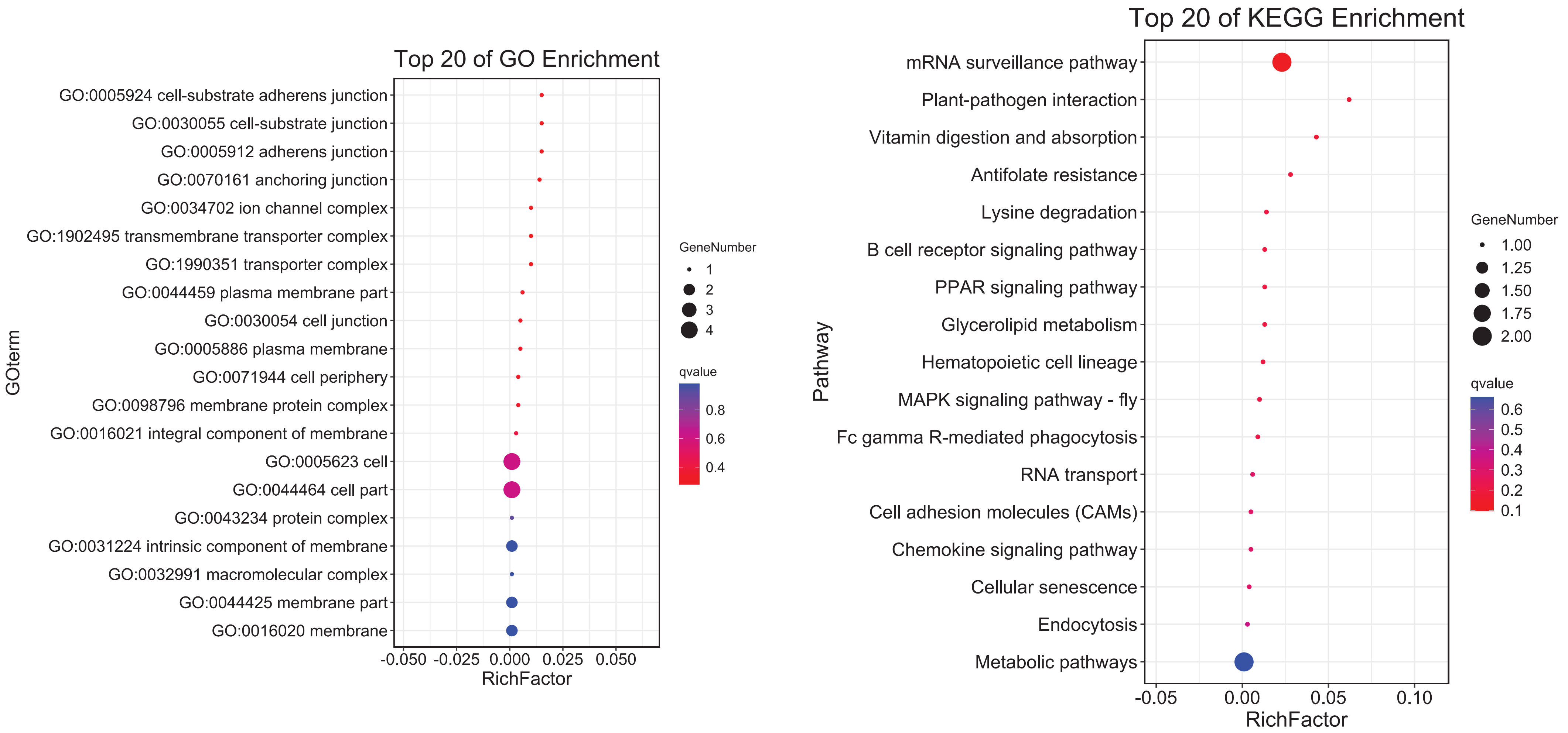

Supplement: Supplementary file 1 [file biology-11-01451-s001.zip › biology-1880822-supplementary.pdf]
